# The proteins of *Fusobacterium* spp. involved in hydrogen sulfide production from L-cysteine

**DOI:** 10.1186/s12866-017-0967-9

**Published:** 2017-03-14

**Authors:** Amina Basic, Madeleine Blomqvist, Gunnar Dahlén, Gunnel Svensäter

**Affiliations:** 1 Department of Oral Microbiology and Immunology, Institute of Odontology, Sahlgrenska Academy, University of Gothenburg, Gothenburg, Sweden; 2 Department of Oral Biology, Institute of Odontology, Malmö University, Malmö, Sweden

**Keywords:** Periodontitis, Hydrogen sulfide, Fusobacterium spp., Enzymes, Bismuth sulfide, Proteomics, 2D gel electrophoresis, LC-MS/MS

## Abstract

**Background:**

Hydrogen sulfide (H_2_S) is a toxic foul-smelling gas produced by subgingival biofilms in patients with periodontal disease and is suggested to be part of the pathogenesis of the disease. We studied the H_2_S-producing protein expression of bacterial strains associated with periodontal disease. Further, we examined the effect of a cysteine-rich growth environment on the synthesis of intracellular enzymes in *F. nucleatum polymorphum* ATCC 10953. The proteins were subjected to one-dimensional (1DE) and two-dimensional (2DE) gel electrophoresis An in-gel activity assay was used to detect the H_2_S-producing enzymes; Sulfide from H_2_S, produced by the enzymes in the gel, reacted with bismuth forming bismuth sulfide, illustrated as brown bands (1D) or spots (2D) in the gel. The discovered proteins were identified with liquid chromatography – tandem mass spectrometry (LC-MS/MS).

**Results:**

Cysteine synthase and proteins involved in the production of the coenzyme pyridoxal 5′phosphate (that catalyzes the production of H_2_S) were frequently found among the discovered enzymes. Interestingly, a higher expression of H_2_S-producing enzymes was detected from bacteria incubated without cysteine prior to the experiment.

**Conclusions:**

Numerous enzymes, identified as cysteine synthase, were involved in the production of H_2_S from cysteine and the expression varied among *Fusobacterium* spp. and strains. No enzymes were detected with the in-gel activity assay among the other periodontitis-associated bacteria tested. The expression of the H_2_S-producing enzymes was dependent on environmental conditions such as cysteine concentration and pH but less dependent on the presence of serum and hemin.

**Electronic supplementary material:**

The online version of this article (doi:10.1186/s12866-017-0967-9) contains supplementary material, which is available to authorized users.

## Background

Oral biofilms differ in composition depending on their niche within the mouth. The biofilms occupying the periodontal pocket, the area between the tooth and the surrounding connective tissue, are usually dominated by Gram-positive, facultative anaerobic bacteria but can undergo a compositional change towards Gram-negative, anaerobic and motile bacteria when oral hygiene is insufficient [1]. The latter biofilms utilize the gingival crevicular fluid as a nutrient source and metabolize proteins, peptides and amino acids to various carboxylic acids and volatile sulfur compounds (VSC). This shift in bacterial ecology along with a host inflammatory response is believed to explain the etiology of periodontal disease where the supportive tissue of teeth is affected by a host immune reaction leading to destruction of alveolar bone (periodontitis).

Hydrogen sulfide (H_2_S) is the most common VSC formed by bacterial degradation of mainly the sulfur-containing amino acid cysteine in the oral cavity. It is a low-molecular weight and volatile gas compound detected in halitosis (bad breath) patients and in periodontal pockets in patients with periodontitis [2–4]. H_2_S is regarded as one of the most toxic metabolites produced in the periodontal pocket. In vitro laboratory studies have shown that H_2_S can damage epithelial cells [5], enhance permeability of the oral mucosa [6] and cause apoptosis of gingival fibroblasts [7]. However, the exact mechanism by which H_2_S exerts its effect on cells is not known. Likewise, the pathogenesis of periodontal disease is poorly understood but it is usually accepted that bacterial metabolites in general, and H_2_S in particular, are of importance in the development and activity of the disease.

Various oral bacterial species are known to be producers of H_2_S. Previous studies by Persson et al. [8] showed that *Porphyromonas endodontalis, Porphyromonas gingivalis, Prevotella intermedia* and *Treponema denticola* were the strongest H_2_S producers when incubated in serum, which contain many of the plasma proteins found in gingival crevicular fluid. In that study, all 163 strains tested were able to produce H_2_S when L-cysteine was used as substrate. Moreover, *Fusobacterium nucleatum* and *Parvimonas micra* were able to generate H_2_S not only from amino acids but also peptides such as glutathione [9, 10]. In our previous in vitro study *Fusobacterium* spp. were the strongest and most rapid producers of H_2_S from L-cysteine, and used the coenzyme pyridoxal 5′phosphate (PLP) [11].

The activity of L-cysteine desulfhydrase, the intracellular enzyme that catalyzes the degradation of cysteine into H_2_S, pyruvate and ammonia, has been shown to vary among different strains of *Fusobacterium* [12]. *F. nucleatum* ATCC 25586 possesses L-cysteine desulfhydrases [13], but these are not the most abundant enzymes involved in the production of H_2_S. The production of a greater amount of H_2_S compared to ammonia and pyruvate suggests that other enzymatic pathways for generation of H_2_S exist. So far, four genes encoding different enzymes involved in H_2_S production have been identified [14–18]. The highest molecular weight enzymes Fn0625 and Fn1419 (47 and 43 kDa respectively) generate H_2_S with pyruvate and ammonia. Fn1220 (the *cdl* gene homologue) is the smallest (33 kDa) but most frequently used enzyme in the formation of H_2_S. It is a L-cysteine desulfhydrase, also known as “L-cysteine lyase”, that catalyzes the β-replacement of L-cysteine giving rise to H_2_S and L-lanthionine [12]. Fn1055 is a 37 kDa protein that catalyzes a reaction that yields H_2_S and L-serine.

In this study we investigated the expression of H_2_S-producing enzymes in 14 bacterial strains associated with periodontal diseases. In addition, we undertook to examine whether a cysteine-rich growth environment induced synthesis of H_2_S producing enzymes and other intracellular enzymes in *F. nucleatum polymorphum* ATCC 10953.

## Methods

### Bacterial strains and culture conditions

Bacterial strains used in this study included type collection strains of *Fusobacterium* spp., *Parvimonas* sp., *Porphyromonas* spp., *Prevotella* spp. and *Treponema* sp. (Table 1), but also fresh clinical isolates from subgingival plaque samples taken from two young adolescents suffering from periodontitis in Ghana [19]. The clinical isolates were typed at Culture Collection University of Gothenburg (CCUG). All strains were recovered on Brucella agar (BBL Microbiology Systems Cockeysville, MD, USA) with 50 ml/l defibrinated horse blood, 20 ml/l hemolyzed human blood and 0.5 mg/l menadione after 5 days incubation under anaerobic conditions (5% CO_2_, 10% H_2_ in N_2_) at 37 °C.

**Table 1:** Bacteria examined for hydrogen sulfide (H_2_S)-producing enzymes, identified with in-gel cysteine digestion and bismuth staining^a^

Species	Subspecies	Strain	Broth
*Fusobacterium canifelinum*		CCUG^b^ 66382	Todd Hewitt
*Fusobacterium necrophorum*	*funduliforme*	ATCC^c^ 51357	Todd Hewitt
*Fusobacterium necrophorum*		CCUG 48192	Todd Hewitt
*Fusobacterium nucleatum*	*polymorphum*	ATCC 10953	Todd Hewitt
*Fusobacterium nucleatum*		OMGS^d^ 3938^e^	Todd Hewitt
*Fusobacterium periodonticum*		ATCC 33693	Todd Hewitt
*Fusobacterium periodonticum*		CCUG 66383	Todd Hewitt
*Parvimonas micra*		ATCC 33270	BHI^f^ + 10% serum
*Porphyromonas endodontalis*		OMGS 1205	BHI + 10% serum
*Porphyromonas gingivalis* (W83)		OMGS 197	BHI
*Porphyromonas gingivalis* (381 F)		CCUG 14449	BHI
*Prevotella intermedia*		ATCC 25611	BHI
*Prevotella tannerae*		ATCC 51259	BHI
*Treponema denticola*		OMGS 3271^g^	Spirochete broth^h^

^a^Bacterial species were grown in broth until OD_600_ of approximately 0.8. After washing and centrifugation, the cells were lysed and the proteins were separated in gel by molecular weight, before staining in bismuth(III)chloride solution containing cysteine. The cysteine-degrading proteins that produced H_2_S were identified in the assay by color change; Sulfide from H_2_S reacted with bismuth and formed bismuth sulfide, a black precipitate. Another set of gels were also stained with conventional Coomassie staining. All experiments were repeated at least once

^b^Culture Collection University of Gothenburg

^c^American Type Culture Collection

^d^Oral Microbiology Gothenburg Sweden

^e^Originally received from Malmö (Badersten 5U)

^f^Brain Heart Infusion broth with 2 mL/L menadione and 10 mL/L hemin

^g^Originally received from Dr R. Ellen, University of Toronto, Toronto, Canada

^h^Dawson JR, Ellen RP. *Tip-oriented adherence to Treponema denticola to fibronectin. Infect Immun*. 1990//;58(12):3924–8

*Fusobacterium* spp. were cultured in Todd Hewitt (TH) broth (Becton Dickinson, Sparks, MD, USA) while *P. micra*, *Porphyromonas* spp. and *Prevotella* spp. were grown in Brain Heart Infusion broth supplemented with menadione (2 ml/l) and hemin (10 ml/l). For growth of *P. micra* and *Prevotella tannerae* the medium was also containing 10% serum.

To investigate the significance of cysteine for the expression of H_2_S-producing enzymes during growth, strains were incubated in the presence of L-cysteine (1 mg/ml) in the appropriate media stated above. For *F. nucleatum* ATCC 10953, the influence of other environmental conditions were tested in TH broth buffered to pH 6, pH 7, pH 8 or TH broth, pH 7.8 supplemented with glutathione (2.5 mg/ml), sodium sulfide (0.46 mg/ml), 5% serum, 50% serum or 50% serum with hemin (10 ml/l). In all cases, the cultures were grown under anaerobic conditions at 37 °C and made in duplicate.

### Preparation of cell extracts (crude enzyme extracts)

Each strain was grown anaerobically in 50 ml culture medium until mid-exponential phase (OD_600_ approximately 0.8) was reached. Cells were harvested by centrifugation (3000 g for 15 min, 4 °C), washed twice in 40 mM Tris pH 9,5 (Sigma-Aldrich Sweden AB, Stockholm Sweden) and resuspended in 1 ml of lysis buffer (5 M urea (Merck KGaA, Darmstadt, Germany), 2 M thiourea (MP Biomedicals, LLC, Illkirch, France), 2% CHAPS (GE Healthcare Bio-Sciences AB, Uppsala, Sweden), 2% sulfobetaine (G-Biosciences, St. Louis, MO, USA), 2 mM tributyl phosphine (Sigma-Aldrich Sweden AB), 40 mM Tris-base pH 9,5 and 2% IPG (GE Healthcare Bio-Sciences AB)). The cell suspensions were shaken gently in room temperature for 1 h with vortexing every 10 min. The extracts were centrifuged (6000 g for 10 min, 4 °C) to remove intact cells and the supernatants were stored separately at −20 °C. The concentration of proteins in the crude enzyme extract was determined with 2-D Quant Kit (GE Healthcare Bio-Sciences AB) following the manufacturer’s instructions.

### One-dimensional gel electrophoresis (1DE)

A 7.5 μl aliquot of the crude enzyme extract (5 – 20 μg protein/sample) was mixed with 2.5 μl of sample buffer NuPAGE LDS (Novex, Carlsbad, CA, USA). Proteins were separated by SDS-PAGE in 4–12% gradient Bis-Tris gels (NuPAGE, Novex) at constant voltage of 200 V for 60 min using NuPAGE SDS MES (Novex) as running buffer. Amersham High-Range Rainbow Molecular Weight Markers (GE Healthcare Bio-Sciences AB) was used as standard.

### Two-dimensional gel electrophoresis (2DE)

Samples of crude enzyme extracts (300 μg protein in 200 μl) were diluted with 130 μl buffer containing 8 M urea, 2% CHAPS, 10 mM dithiothreitol (GE Healthcare Bio-Sciences AB), 2% IPG and 0.01% bromophenol blue and placed in re-swelling cassettes under Immobiline dry gel (IPG) Strips (pH 4–7, 18 cm; GE Healthcare Bio-Sciences AB). The loading and rehydration of IPG strips took place at room temperature for 24 h under silicone oil. Isoelectric focusing was conducted using Multiphor II (GE Healthcare Bio-Sciences AB) with supply of cooling water at 15 °C. Isoelectric focusing was initiated at 150 V for 1 h, the voltage increased gradually during 18 h to 1200 V and maintained at 3500 V for 20 h. After focusing, the strips were stored at −80 °C. Before separation of proteins in the second dimension, the IPG strips were equilibrated first in 50 mM Tris–HCl (pH 6.8), 2% SDS, 26% glycerol and 16 mM dithiothreitol for 15 min and then for another 15 min in the same buffer but containing 250 mM iodoacetamide (GE Healthcare Bio-Sciences AB) and 0.005% bromophenol blue instead of dithiothreitol. The IPG strips were embedded, using 0.5% (w/v) molten agarose, on top of 14% polyacrylamide gels (0.38 M Tris buffer pH 8.8, 14% Bis-acrylamide (Bio-Rad Laboratories, Sundbyberg, Sweden), 0.1% SDS, 4.6% glycerol, 0.05% TEMED (Bio-Rad Laboratories) and 0.05% ammonium persulfate (Bio-Rad Laboratories)). SDS-PAGE was run in PROTEAN II xi Cell (Bio-Rad Laboratories) at constant current (19 mA) overnight with running buffer containing 50 mM Tris (pH 8.3), 0.1% SDS and 0.384 M glycine.

### Detection of H_2_S-producing enzymes

The enzymes degrading L-cysteine and forming H_2_S were detected through precipitation of bismuth sulfide using an in-gel activity assay, essentially as described previously [12, 16]. H_2_S-producing enzymes appeared as brown to black bands in the 1DE gels and as spots in the 2DE gels. Before bismuth staining, the gels were subjected to a renaturation process where SDS was removed and replaced with nonionic detergents. The renaturation took place during gentle shaking at 4 °C with the following solutions: (i) 25 mM triethanolamine-HCl pH 8.0, 0.05% SDS and 0.5% Triton-X-100 for 1 h; (ii) 25 mM triethanolamine-HCl pH 8.0, 0.5% Triton-X-100 and 0.5% Lubrol PX for 2 × 1 h; (iii) 25 mM triethanolamine-HCl pH 7.0 and 0.5% Lubrol PX for 2 × 0.5 h. For activity staining, the gels were incubated in 100 mM triethanolamine-HCl pH 7.6, 10 μM pyridoxal 5-phosphate monohydrate (VWR, Stockholm, Sweden), 0.5 or 1.0 mM bismuth trichloride (Fisher Scientific GTF AB, Gothenburg, Sweden), 10 mM EDTA (Sigma-Aldrich Sweden AB) and 5 or 20 mM L-cysteine (Sigma-Aldrich Sweden AB) at 37 °C for 2 h. All the activity assays, including both 1DE and 2DE gels, were performed at least twice, with double sets of gels for staining with bismuth and Coomassie staining.

### Coomassie and silver staining

Before staining, 1DE gels were fixed in 40% ethanol and 2% acetic acid for 1 h, and 2DE gels in 40% ethanol and 5% acetic acid for 0.5 h. Gels were stained with 16% Coomassie brilliant blue G colloidal concentrate (Sigma-Aldrich Sweden AB) in 20% ethanol overnight at room temperature. After rinsing in 5% acetic acid and 25% ethanol for 1 min, gels were destained in 25% ethanol for 1–3 h and washed with ultra-high quality water. The 2DE gels were also stained with silver according to the protocol of the manufacturer (GE Healthcare Bio-Sciences AB).

### Identification of proteins by mass spectrometry

Protein spots of interest were excised manually from Coomassie brilliant blue stained 2DE gels of crude cell extract and subjected to LC-MS/MS as described previously [20]. Briefly, proteins in gels were reduced with dithiothreitol, alkylated with iodoacetamide and then digested with trypsin. Tryptic peptides were separated and analyzed by mass spectrometry. The peaks were later identified by creating Mascot Generic Files and by database searching using Matrix science web server (www.matrixscience.com).

## Results

### H_2_S-producing enzymes among bacterial strains

**Fig. 1:**
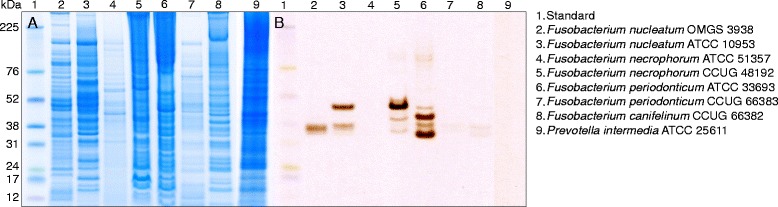
The protein expression of different bacterial strains grown in broth without cysteine was examined with gel electrophoresis. With Coomassie staining (**a**) all proteins were stained. However, the in-gel activity assay with bismuth staining (**b**) only detected the proteins that produced hydrogen sulfide (H_2_S) from cysteine. Sulfide from H_2_S reacted with bismuth and formed bismuth sulfide, a brown to black precipitate

Cell extracts of 14 strains of bacteria associated with periodontitis (Table 1) were screened for H_2_S-producing enzymes with in-gel activity assay after renaturation. All *Fusobacterium* spp. except *F. necrophorum* ATCC 51357 had enzymes producing H_2_S, detected as brownish bands on the gels (Fig. 1). *F. nucleatum* OMGS 3938 displayed one band around 37 kDa while *F. nucleatum* ATCC 10953 showed 37 kDa and 47 kDa enzymes illustrating differences within the same subspecies. The *F. necrophorum* strain that showed activity had three bands with sizes around 47, 43 and 33 kDa. *F. periodonticum* ATCC 33693 displayed bands at 47, 43, 37 and 33 kDa and the remaining two clinical isolates of *Fusobacterium* spp. had low activity at 37 and 33 kDa (Fig. 1). Other bacterial species associated with periodontitis such as *P. gingivalis, P. intermedia, P. micra, P. tannerae* and *T. denticola* were also examined with this method and no H_2_S-producing enzymes could be detected.

**Fig. 2:**
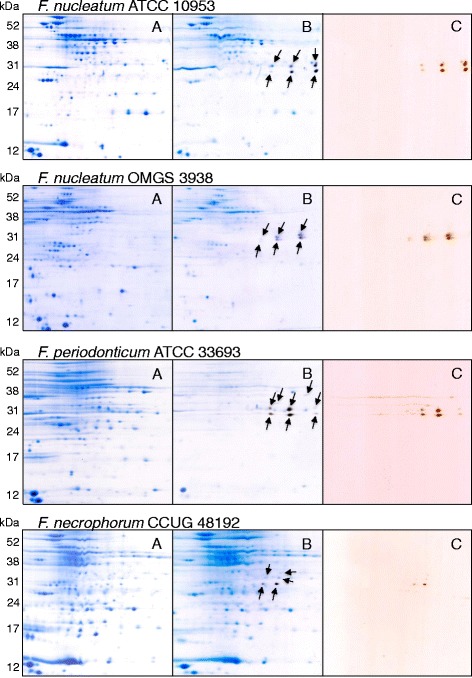
Two-dimensional gel-electrophoresis of proteins extracted from different *Fusobacterium* spp. The bacteria were grown in Todd Hewitt broth without cysteine prior to protein extraction and separation. One gel was stained with Coomassie *blue* (**a**) for protein detection and extraction and another with in-gel activity bismuth staining (**c**) for detection of proteins producing H_2_S. Bismuth reacts with sulfide and produces a precipitate, bismuth sulfide, shown as brown spots in the gel. After bismuth staining the same gel was stained with Coomassie *blue* (**b**). As illustrated in the figure the protein expression of the subspecies differed

Cell extracts of selected *Fusobacterium* spp., were also separated by isoelectric focusing and molecular weight in two-dimensional gel electrophoresis. The gels were, after protein separation, stained with silver staining for better resolution or Coomassie staining for protein extraction and identification. The total protein expression for different subspecies and strains of *Fusobacterium* differed in both pattern and intensity (Fig. 2). The H_2_S-producing enzymes were colored in the bismuth solution and extracted from the Coomassie stained gels. The most frequently detected enzymes were identified as cysteine synthase, involved in cysteine metabolism. Also a protein involved in the biosynthesis of the coenzyme pyridoxal phosphate was identified (Additional file 1: Table S1).

### The effect of environmental conditions on enzyme expression in Fusobacterium spp.

**Fig. 3:**
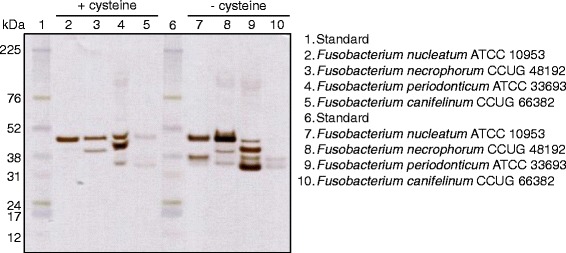
A protein separation by molecular weight of enzymes from *Fusobacterium* spp. Bacteria were grown in broth with and without cysteine prior to protein extraction and in-gel activity assay that stained the proteins that produced H_2_S

To investigate the significance of cysteine for the expression of H_2_S-producing enzymes, all strains were grown in the presence of L-cysteine (1 mg/ml) in appropriate growth media. A difference in protein expression, both with regard to the number of bands and the intensity of the color, between bacteria grown in broth with and without cysteine prior to the experiment was seen on the bismuth-stained gels (Figs. 1 and 3). When bacteria were grown in broth without cysteine, additional and stronger bands appeared on the gels. For *F. nucleatum* ATCC 10953 the 47 kDa band was shown in both environments while the 37 kDa band was only clearly seen after growth in broth without cysteine. Similarly, the band with the enzyme of the smallest size, around 33 kDa was seen for *F. necrophorum* CCUG 48192 without cysteine. In addition, the largest enzyme (47 kDa) was enhanced without cysteine. For *F. periodonticum* ATCC 33693 a fourth band was seen (37 kDa) without cysteine and the smallest enzyme (around 33 kDa) was enhanced. The clinical isolate *F. canifelinum* CCUG 66382 showed lowest activity and had bands of the size 47 kDa and 33 kDa when grown with cysteine and 37 kDa and 33 kDa without cysteine.

**Fig. 4:**
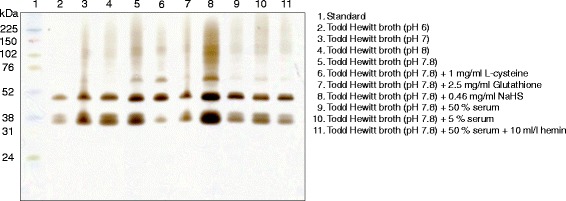
The effect of the environmental conditions on the activity of H_2_S-producing enzymes was tested for *Fusobacterium nucleatum polymorphum* ATCC 10953. The bacteria were grown in different broths before protein extraction and 1D gel electrophoresis followed by in-gel activity assay

*F. nucleatum polymorphum* ATCC 10953 was selected for further studies on the influence of environmental conditions on expression of H_2_S-producing enzymes and therefore incubated in TH broth buffered to pH 6, pH 7, pH 8 or TH broth supplemented with glutathione, NaHS, 5% serum, 50% serum or 50% serum with hemin (Fig. 4). All tested modifications of the broth resulted in at least two clear bands on the bismuth stained gels. When the bacteria were incubated in broth containing sodium hydrosulfide (NaHS) the strongest bismuth sulfide precipitation was detected and 2–3 clear bands were shown. Also bacteria incubated in TH broth without any additives produced strong bands compared with all but NaHS. When L-cysteine was added the bands with lower molecular weight values produced less H_2_S, compared with the broth without any additives. The readers should note that a higher concentration of bismuth trichloride and L-cysteine was used here (Fig. 4) compared to the initial experiments (Fig. 3), which may explain the band with lower molecular weight seen in Fig. 4 but not initially in Fig. 3. The different pH values tested, all displaying the same bands, showed minor trends toward a more pronounced staining intensity when incubated at a higher pH. A higher enzymatic activity was not seen when serum, glutathione or hemin were added to the broth.

### The cellular response to cysteine-rich environment

**Fig. 5:**
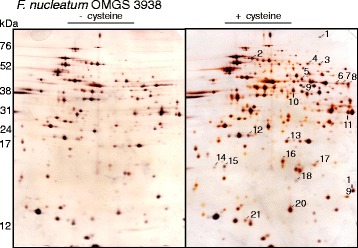
Two-dimensional gel-electrophoresis of *Fusobacterium* OMGS 3938 grown in Todd Hewitt broth with and without cysteine prior to protein extraction and separation. Silver staining was used to detect the proteins. The proteins enhanced when the bacteria was grown in cysteine, compared to the protein expression when they were grown without cysteine, were extracted (*line*) and identified (Table 2)

Differences in protein expression between bacteria grown in cysteine-rich and poor broth were also examined by protein extraction of spots enhanced in the gels where the bacteria were grown in cysteine-rich broth (Fig. 5). The extracted proteins, identified with LS-MS/MS (Table 2), were glycolytic proteins, proteins involved in butyrate metabolism and oxidoreductase. Also a protein involved in pyridoxal 5′phosphate biosynthesis was identified (a coenzyme for the degradation of cysteine and production of H_2_S).

**Table 2:** Proteins of *Fusobacterium nucleatum* enhanced when incubated in cysteine-rich broth prior to protein extraction*

Spot no.	Protein	Protein function
1	Glyceraldehyde-3-phosphate dehydrogenase^a, b^	Glycolytic protein
2	Bifunctional penicillin tolerance protein LytB/ribosomal protein S1 RpsA^a^	Translation
3, 4	Pyruvate kinase^b, c^	Glycolytic protein
5	Recombination protein A^d^ /Histidyl-tRNA synthetase^e, f^	DNA repair/histidyl-tRNA aminoacylation
6, 8	Acetate kinase^b, g^	Acetyl-CoA biosynthetic process
7	Electron transfer flavoprotein subunit alpha^b^	Electron carrier activity
9	Phosphoglycerate kinase^b, h^	Glycolytic protein
10	Zn-dependent alcohol dehydrogenase and related dehydrogenase^b^	Oxidoreductase, zinc ion binding
11	Pyridoxal biosynthesis lyase PdxS^b^	Pyridoxal 5′-phosphate
12	Butyrate-acetoacetate CoA-transferase subunit B^b^	Butyrate metabolism
13	Acetoacetate: butyrate/acetate coenzyme A transferase^b^	Butyrate metabolism
14	Iron-sulfur cluster-binding protein^b^	Iron and sulphur binding
15	(S)-2-hydroxy-acid oxidase chain D^i^ /glycolate oxidase, subunit GlcD^g^	Oxidoreductase
16, 17	Rubrerythrin^a^	Oxidoreductase, iron ion binding
18	PTS-system, N-acetylglucosamine-specific IIA component^b^	Phosphotransferase system
19	Mannose-1-phosphate guanylyl transferase (GDP)^b^	GDP-mannose biosynthetic process, lipopolysaccharide biosynthetic process
20	Translation initiation inhibitor^b^	Deaminase activity
21	Anti-sigma F factor antagonist^b^	Regulation of transcription

*Fusobacterium nucleatum OMGS 3938 was incubated in Todd Hewitt broth with and without cysteine. The spots that were enhanced when incubated in cysteine-rich broth were extracted for identification with LC- MS/MS

^a^ *Fusobacterium nucleatum* subsp. *polymorphum* ATCC 10953

^b^ *Fusobacterium nucleatum* subsp. *nucleatum* ATCC 25586

^c^ *Fusobacterium* sp. 7_1

^d^ *Fusobacterium nucleatum* subsp. *nucleatum* ATCC 23726

^e^ *Fusobacterium* sp. 4_1_13

^f^ *Fusobacterium nucleatum* ChDC F128

^g^ *Fusobacterium periodonticum* ATCC 33693

^h^ *Desulfosporosinus* sp. OT

^i^ *Fusobacterium nucelatum* subsp. *vicentii* ATCC 49256

## Discussion

The production of H_2_S is complex and involves different enzymatic pathways for different bacterial species and strains. The literature on this subject is rather sparse as opposed to the production of eukaryotic cells, where H_2_S is produced by three PLP dependent enzymes; cystathionine β-synthase, cystathionine γ-lyase and 3-mercaptopyruvate sulfurtransferase, that use L-cysteine as their principle substrate [21]. The bacterial production of H_2_S is mainly due to the degradation of the sulfur-containing amino acid cysteine and results in different metabolic end products depending on the enzymes participating. One common cysteine degradation pathway involves the PLP dependent L-cysteine desulfhydrases, including α, β-elimination activity, that results in the production of H_2_S, pyruvate and ammonia [22, 23]. L-cysteine desulfhydrases have been identified in many oral bacterial species and are known to be encoded by the *cdl* gene in *F. nucleatum* [14], the *hly* gene in *T. denticola* [24] and the *lcs* gene in *P. intermedia* [25]. Moreover, *Streptococcus anginosus* and *S. intermedius* are capable to produce H_2_S from L-cysteine using a cystathionase, encoded by the *lcd* gene, that uses L-cystathionine as well as cysteine as substrate [26–28]. In the current study, the in-gel activity assay for detection of H_2_S-producing enzymes revealed a variety of enzymes with molecular weights between 30 and 50 kDa in *F. nucleatum*, *F. necrophorum* and *F. periodonticum*. The sizes of these enzymes are in line with the desulfhydrases previously reported for *F. nucleatum* ATCC 25586; 33 kDa (Fn1220, *cdl*), 37 kDa (Fn1055), 43 kDa (Fn1419) and 47 kDa (Fn0625) [18]. It is therefore tempting to suggest that similar desulfhydrases are also involved in H_2_S-production in *F. necrophorum* and *F. periodonticum*.

When in-gel activity assays were used to investigate the H_2_S-producing enzyme profile in cell extracts of *P. gingivalis*, *P. intermedia, P. micra*, *P. tannerae* and *T. denticola* no H_2_S-producing protein bands could be detected despite previous reports of the ability to produce H_2_S for these bacterial species [8, 11]. The lack of activity may be due to several factors such as strain differences, suboptimal conditions for enzyme reactivation after SDS-PAGE or a lower affinity of the enzyme to bind cysteine. The reported K_m_-values of enzymes extracted from *T. denticola* are high compared to *Fusobacterium* spp. [14, 17], which suggests that the method used in this study is not sensitive enough to detect the enzymes with lower affinity to L-cysteine.

The most prominent H_2_S-producing enzymes in *F. nucleatum*, *F. necrophorum* and *F. periodonticum* were found around 30 kDa on 2DE gels (Fig. 2). The majority of protein spots exhibiting precipitates of bismuth sulfide were excised from 2DE-gels and subjected to mass spectrometric analysis. The results revealed that all proteins could be allocated to cysteine synthases. Further analysis of the amino acid sequences of cysteine synthase from the three species showed almost complete homologies with the sequence reported for *cdl* (Fn1220) in *F. nucleatum*. Yoshida and coworkers reported approximately 40% identity of the H_2_S producing gene Fn1220 from *F. nucleatum* to cysteine synthases A and B in *E. coli* and suggested that both these enzymes may catalyze both of the reactions that result in the production of H_2_S and L-lantionine and of L-cysteine and acetate respectively [15]. One can therefore assume that H_2_S production in different species of *Fusobacterium* is the result of the condensation of cysteine molecules with lanthionine as a byproduct.

In this study, enzymatic H_2_S-producing activity was detected for *F. necrophorum* CCUG 48192 but not for strain ATCC 51357 (Fig. 1). This confirms results from previous reports of the differences in H_2_S producing capacity among different strains of *Fusobacterium* [12, 16]. However, the variance seen does not seem to be something unique for this genus. Similar variations in H_2_S production have been reported for different subspecies of S*treptococcus* [27, 28]*.* L-cysteine desulfhydrase activity for some *Fusobacterium* spp. and L-cysteine lyase activity for other strains adds on the complexity by the diverse enzymes being active under aerobic and anaerobic conditions [12].

The expression of H_2_S-producing enzymes was not significantly affected by the presence of serum proteins or the pH of growth medium (Fig. 4). However, as illustrated in Figs. 3 and 4, lower expression of H_2_S-producing enzymes was demonstrated for *F. nucleatum*, *F. necrophorum* and *F. periodonticum* when cells were grown in broth supplemented with cysteine compared to without cysteine. These results indicate cysteine-mediated down-regulation of these enzymes in the genus *Fusobacterium*. In all species, the enzyme expression mostly affected was that with the lowest molecular weight, which probably correspond to Fn1220. Of interest is that the Fn1220 enzyme is known to exhibit the highest H_2_S-producing activity and is responsible for more than 85% of the H_2_S production in *F. nucleatum* [18]. In addition, the ability of the enzyme to degrade cysteine is inversely related to cysteine concentration. When comparing different concentrations of cysteine as substrate, a higher sulfide production was observed at 0.5 mM L-cysteine-HCl than at 2 mM and 6 mM, which suggests that desulfuration is inhibited by the excess of substrate also on the enzyme activity level [13]. This might be indicative of a mechanism that supports bacterial survival and limits production of toxic H_2_S in cysteine-rich environments.

Proteomics has recently been reviewed [29]. Despite some drawbacks with the method, such as that some proteins are excluded because of very high and low isoelectric point and molecular weight, a majority of the proteins expressed by bacteria that have been exposed to changed environmental factors can be studied [30]. When *F. nucleatum* OMGS 3938 was grown in the presence of cysteine more than one hundred proteins were differently expressed compared to cells grown without cysteine. The observed down-regulation of H_2_S-producing enzymes in cells grown in cysteine-rich environment, as previously demonstrated by SDS-PAGE followed by in-gel activity staining (Fig. 3), was supported by the observation of a higher expression on 2DE gels from cells grown in the absence of cysteine (data not shown). Twenty-one abundant protein spots exhibited more than a two-fold increase in optical intensity and these were subjected to identification with LC-MS/MS. Many of these proteins were identified as glycolytic enzymes, oxidoreductases or proteins involved in the butyrate metabolism (Table 2). These results suggest that the primary metabolic pathway for carbohydrate metabolism is activated during growth in a cysteine-rich environment. Of interest is that nine of the up-regulated proteins identified in this study (1, 6, 7, 8, 10, 11, 12, 13, 14 in Table 2) were down-regulated when anaerobically grown cells of *F. nucleatum* were exposed to oxygen [31]. This confirms the reducing potential of cysteine and thus the avoiding of oxidative stress. Cysteine has many functions besides being a substrate in the formation of H_2_S; it contributes to a more anaerobic environment by reduction.

## Conclusions

Periodontal disease is defined as an infectious disease but the role of the biofilm and the host-parasite interaction is still unknown. The bacterial metabolism and the net effect of a biofilm is of importance in the understanding of the mechanisms involved where biofilms are contributing to disease development. In this study we focused on bacterial production of H_2_S from cysteine. Numerous enzymes, identified as cysteine synthase, were involved in the production of H_2_S from cysteine and the expression varied among *Fusobacterium* spp. and strains. No enzymes were detected with the in-gel activity assay among the other periodontitis-associated bacteria tested. The expression of the H_2_S-producing enzymes was dependent on environmental conditions such as cysteine concentration and pH but less dependent on the presence of serum and hemin. Knowledge of H_2_S-production and the possible affect it may have on host cells is needed to elucidate its potential role in the pathogenesis of periodontal disease.

## Additional file

Additional file 1: Table S1. Identification of proteins of *Fusobacterium* spp. involved in hydrogen sulfide (H_2_S) production, detected with in-gel cysteine digestion and bismuth staining*. (DOC 47 kb)

## Abbreviations

1DE: One dimensional gel electrophoresis
2DE: Two dimensional gel electrophoresis
H_2_S: Hydrogen sulfide
LC-MS/MS: Liquid chromatography – tandem mass spectrometry
PLP: Pyridoxal 5′phosphate
VSC: Volatile sulfur compounds
