# Supplementary material for: The proteins of Fusobacterium spp. involved in hydrogen sulfide production from L-cysteine
Source: BMC Microbiol. 2017 Mar 14;17:61. doi: 10.1186/s12866-017-0967-9 (PMC5348791; doi:10.1186/s12866-017-0967-9)
Supplement: Additional file 1: Table S1. — Identification of proteins of Fusobacterium spp. involved in hydrogen sulfide (H2S) production, detected with in-gel cysteine digestion and bismuth staining*. (DOC 47 kb) [file 12866_2017_967_MOESM1_ESM.doc]

| **Species** | **Strain** | **Spot no.** | **Protein** | **Protein function** |
| --- | --- | --- | --- | --- |
| *F. necrophorum* | CCUG[[1]](#footnote-2) 48192 | 1 | Cysteine synthase[[2]](#footnote-3) | Cysteine metabolism |
|  |  | 2 | No significant Hits |  |
| *F. nucleatum polymorphum* | ATCC[[3]](#footnote-4) 10953 | 1 | Cysteine synthase[[4]](#footnote-5) / Pyridoxine biosynthesis protein[[5]](#footnote-6) | Cysteine metabolism / Biosynthesis of pyridoxine |
|  |  | 2 | Cysteine synthase^d^/ 3-hydroxybutyryl-CoA dehydrogenase^b^ / | Cysteine metabolism / Butyrate metabolism |
|  |  | 3 | Cysteine synthase^db^ | Cysteine metabolism |
|  |  | 4 | Cysteine synthase^d^ / 2,5-diketo-D-gluconic acid reductase^b^ | Cysteine metabolism / Oxidoreductase |
| *F. nucleatum* | OMGS[[6]](#footnote-7) 3938[[7]](#footnote-8) | 1 | Cysteine synthase A^b^ / Cysteine synthase^b^ | Cysteine metabolism |
|  |  | 2 | Cysteine synthase^b^ / Pyridoxine biosynthesis protein^e^ | Cysteine metabolism / Biosynthesis of pyridoxine |
| *F. periodonticum* | ATCC 33693 | 1 | Pyridoxal biosynthesis lyase[[8]](#footnote-9) / Cysteine synthase^h^ | Biosynthesis of pyridoxine / Cysteine metabolism |
|  |  | 2 | Cysteine synthase^h^ / 30S ribosomal protein S2^h^ | Cysteine metabolism / Translation |
|  |  | 3 | Cysteine synthase^h^ / Pyridoxal biosynthesis lyase^h^ | Cysteine metabolism / Biosynthesis of pyridoxine |
|  |  | 4 | Cysteine synthase^h^ / 30S ribosomal protein S2^h^ | Cysteine metabolism / Translation |

**Table S1.** Identification of proteins of *Fusobacterium* spp. involved in hydrogen sulfide (H_2_S) production, detected with in-gel cysteine digestion and bismuth staining *

***** *Fusobacterium* spp. incubated in Todd Hewitt broth without (-) cysteine prior to protein extraction and 2D gel electrophoresis. The gels were colored with bismuth solution for identification of proteins producing H_2_S and with coomassie staining followed with protein extraction and identification with LC- MS/MS.

1. Culture Collection University of Gothenburg [↑](#footnote-ref-2)
2. *Fusobacterium nucleatum* [↑](#footnote-ref-3)
3. American Type Culture Collection [↑](#footnote-ref-4)
4. *Fusobacterium* [↑](#footnote-ref-5)
5. *Fusobacterium nucelatum polymorphum ATCC 10953* [↑](#footnote-ref-6)
6. Oral Microbiology Gothenburg Sweden [↑](#footnote-ref-7)
7. Originally recieved from Malmö (Badersten 5U) [↑](#footnote-ref-8)
8. *Fusobacterium periodonticum* [↑](#footnote-ref-9)
